# Editorial: Insights in gastroenterology: 2021

**DOI:** 10.3389/fmed.2022.1008157

**Published:** 2022-09-08

**Authors:** Angel Lanas, Guillermo García-Rayado

**Affiliations:** ^1^Department of Medicine, University of Zaragoza, Zaragoza, Spain; ^2^Biomedical Research Networking Center in Hepatic and Digestive Diseases (CIBERehd), Madrid, Spain; ^3^Aragón Health Research Institute (IIS Aragón), Zaragoza, Spain; ^4^Service of Digestive Diseases, University Clinic Hospital Lozano Blesa, Zaragoza, Spain

**Keywords:** colorectal cancer, gastric cancer, non-alcoholic fatty liver disease, functional defecation disorders, cancer, functional diseases

*Insights in Gastroenterology: 2021* was a Research Topic with the objective of highlighting the latest advances and future challenges in the field of gastroenterology research. Gastroenterology research has a very wide spectrum that includes different pathologies, from diseases of the digestive tract to liver and pancreatic pathologies. This editorial focuses on novel developments in non-alcoholic fatty liver disease (NAFLD), early diagnosis of colorectal cancer (CRC) and gastric cancer (GC) and in functional defecation disorders (FDD) which reflect the manuscript submitted and published within the topic.

The prevalence of NAFLD is increasingly worryingly (1) and it is currently the most common form of liver disease and the leading cause of liver transplantation in some Western countries as the United States (2). For this reason, advances in understanding the pathogenesis and epidemiology as well as in the diagnosis and treatment of this disease are greatly needed. In the field of epidemiology, previous studies have shown that there is a racial/ethnic disparity with NAFLD (3). Furthermore, studies on the influence of gender on the development of NAFLD show conflicting conclusions. In this line, Shaheen et al. analyzed data for 3.292 citizens of United States using the *National Health and Nutrition Examination Survey* to examine the gender role in the racial/ethnic difference in NAFLD. They found an overall prevalence of NAFLD of 47.9%, with the highest prevalence of severe NAFLD among Mexican Americans (46%) and lowest among non-Hispanic Blacks (22.7%) (*p* < 0.05). In the adjusted model, relative to non-Hispanic White population, Mexican American were more than twice as likely to have severe NAFLD (AOR = 2.4, 95% CI = 1.4–4.2, *p* < 0.05), whereas black population was significantly less likely to have severe NAFLD (AOR = 0.5, 95% CI = 0.4–0.7, *p* < 0.05). However, these associations were modified when they were stratified by sex. The increased AOR of NAFLD found for Mexican Americans was only observed for males. In addition, related risk factors shown in the results of the study differed by sex. In view of these results, the pathophysiological pathways of NAFLD could be different according to gender and race, aspects that are not frequently taking into consideration and that merits further research. Besides, according to the study findings, screening and interventions that target Mexican Americans must be prioritized.

In the field of treatment of NAFLD, a diet regimen and physical exercise remain the cornerstone of treatment, but some pharmacological therapies have shown some benefits. For example, silybin, the active compound of the silymarin, has shown to improve metabolic parameters involved in NAFLD (4). On the other hand, some genetics variants of PNPLA3, TM6SF2 and MBOAT7 genes identified in genome-wide and exome-wide association studies in patients with fatty livers, fatty livers, seem to be linked to a faster worsening of NAFLD (5). Therefore, a few studies have assessed the influence of these genetic variants on the response to treatment. Dallio et al. conducted the first randomized controlled trial to evaluate the effect of a silybin-phospholipids complex during 6 months in NAFLD patients carrying either wild type (controls) or mutated PNPLA3, TM6SF2 and MBOAT7 genes. The wild type treated group showed a significant improvement of some metabolic parameter like glycemia, ALT or thiobarbituric acid reactive substance whereas there were no changes in patients with PNPLA3, TM6SF2 and MBOAT7 genetic variants. Hence, this study represents an advance toward a tailored treatment of patients with NAFLD. Next trials should analyse the influence of these and other genetic variants in the different treatments of NAFLD including homozygosis or heterozygosis genotype and which is more important, new clinical trials with larger sample size and longer duration of therapy should be able to investigate not only the effect of any intervention in metabolic parameters but also on possible histological changes.

When men and woman are considered together, CRC is the most frequent cancer in western countries (6). Many countries have screening systems of CRC but theses screening programs have some limitations as the high false-positive rate of fecal occult blood test (7). On the other hand, the pathogenesis of CRC is associated with systemic inflammation and some inflammatory blood analytical markers have been used to estimate the prognosis of CRC. However, its usefulness for diagnosis of CRC has been little studied. Hernández-Ainsa et al. carried out a retrospective case-control study to analyze previous inflammatory blood markers and a new marker (named NP/LHb = [neutrophils x platelets]/[lymphocytes x hemoglobin]) as a diagnostic tool. In the CRC group, they evaluated blood analytical markers collected at time of the diagnosis but also those obtained before (a median of 6 months) the diagnosis of CRC was made. NP/LHb showed a specificity of 92.06%, a positive predictive value of 87.50% and the best area under the curve (AUC: 0.78) of all inflammatory markers to diagnose CRC. In addition, NP/LHb levels were highest at time of diagnosis compared with levels in the previous 6 months and in the control group. This is the first study that assesses inflammatory markers at two different times and that shows a progressive increase of inflammatory markers obtained easily from a single hemogram. Besides, due to the high positive predictive value obtained, NP/LHb could be used together with fecal occult blood tests to improve the accuracy of CRC screening. Authors propose that the fecal occult blood test could be performed first, and if it is positive the NP/LHb index should be determined. If this new index was also positive, a colonoscopy should be prioritized because the risk of CRC in these patients should be higher than those with a single positive fecal test. This hypothesis requires validation in new studies.

GC is the fifth most frequent cancer and the third leading cause of cancer mortality (8). Gastric atrophy and intestinal metaplasia are included within the spectrum of atrophic gastritis (AG). Most gastric epithelial cancers arise from AG (9). Many studies on AG have been carried out in the last decade, however no systematic analysis of scientific research on this topic has been performed. A well-performed systematic analysis to evaluate the research in this field can be useful in guiding researchers and optimizing their work. Zhang et al. carried out a deep bibliometric analysis of AG from 2011 to 2021 that included 1.432 publications. China (that has one of the higher incidences of GC) was the most productive country regarding total number of publications followed by United States and Japan. Nevertheless, the analysis of centrality reveals more collaborative research and influential papers in the United States compared to China and other Asian countries. The institution (*Vanderbilt University*) and the author (*James R Golderning*) with the highest number of publications and cooperations also come from the United States. It is suggested that China and other Asian countries should expand their collaborative research and their connections especially with United States and Europe if they want to become more influential. On the other hand, the analysis of the burst keywords in the last 5 years highlights words as chief cell, spasmolytic polypeptide-expressing metaplasia (SPEM) and stem cells. SPEM may be a potential neoplastic lesion that could evolve to intestinal metaplasia (10) but it is a subject of debate nowadays. In view of these results, exploring the evolution and origin of SPEM should be a direction of research on AG in the next years.

Finally, FDD are characterized by constipation symptoms with paradoxical contraction or inadequate relaxation of the pelvic floor muscles and/or no adequate propulsive forces during attempted defecation (11, 12). FDD are a common cause of distress, deterioration of the quality of life and an important healthcare burden (12). However, its pathophysiological mechanisms are not fully understood. In this sense, *Pucciani F*. and *Trafeli M* tried to analyze the rectoanal inhibitory reflex (RAIR) in 58 patients with FDD (13). They found an incomplete/short duration of RAIR (the average relaxation was 74% compared to 92% in their control group) and an excessive contraction/duration of rectoanal excitatory reflex (an average of 13 mmHG pressure increase). The authors concluded that the sampling reflex is impaired in patients with FDD. However, Huizinga et al. from *McMaster University* of Canada make a critical reading of the article of *Pucciani F*. and a review of the pathophysiology of distal colon motor coordination. They consider that alterations found by *Pucciani F*. are normal according to international standards (14) and that these manometric parameters of a normal RAIR do not reveal the pathogenesis of obstructive defecation. In addition, Huizinga et al. make a concise but complete review about the value of assessing the RAIR, the relaxation and dyssynergia and the coloanal reflex. It follows from this review that more efforts should be made for the standardization of findings of anorectal manometry and that future research should pay attention to coloanal reflex. Coloanal reflex is very important for complete defecation because it can relax both anal sphincters and alterations of this reflex can be the primary pathogenesis of obstructed defecation. Therefore, they conclude that in addition to RAIR, coloanal reflex should be explored in further studies.

## Author contributions

AL designed and revised the editorial of the Research Topic. GG-R drafted the Editorial of the Research Topic. Both authors contributed to the article and approved the submitted version.

## Funding

GG-R was supported by grant from the Instituto de Salud Carlos III [Juan Rodés grant JR 21/00012].

## Conflict of interest

The authors declare that the research was conducted in the absence of any commercial or financial relationships that could be construed as a potential conflict of interest.

## Publisher's note

All claims expressed in this article are solely those of the authors and do not necessarily represent those of their affiliated organizations, or those of the publisher, the editors and the reviewers. Any product that may be evaluated in this article, or claim that may be made by its manufacturer, is not guaranteed or endorsed by the publisher.

## Abbreviations

AG: atrophic gastritis
CRC: colorectal cancer
FDD: functional defecation disorders
GC: gastric cancer
NAFLD: non-alcoholic fatty liver disease
NP/LHb: [neutrophils x platelets]/[lymphocytes x hemoglobin]
RAIR: rectoanal inhibitory reflex
SPEM: spasmolytic polypeptide-expressing metaplasia.
